# Loss of Optineurin *In Vivo* Results in Elevated Cell Death and Alters Axonal Trafficking Dynamics

**DOI:** 10.1371/journal.pone.0109922

**Published:** 2014-10-16

**Authors:** Jeremiah D. Paulus, Brian A. Link

**Affiliations:** Department of Cell Biology, Neurobiology and Anatomy, Medical College of Wisconsin, Milwaukee, WI, United States of America; Casey Eye Institute, United States of America

## Abstract

Mutations in Optineurin have been associated with ALS, glaucoma, and Paget’s disease of bone in humans, but little is known about how these mutations contribute to disease. Most of the cellular consequences of Optineurin loss have come from *in vitro* studies, and it remains unclear whether these same defects would be seen *in vivo*. To answer this question, we assessed the cellular consequences of Optineurin loss in zebrafish embryos to determine if they showed the same defects as have been described in the *in vitro* studies. We found that loss of Optineurin resulted in increased cell death, as well as subtle cell morphology, cell migration and vesicle trafficking defects. However, unlike experiments on cells in culture, we found no indication that the Golgi apparatus was disrupted or that NF-κB target genes were upregulated. Therefore, we conclude that *in vivo* loss of Optineurin shows some, but not all, of the defects seen in *in vitro* work.

## Introduction

Optineurin (OPTN) has been associated with a number of different diseases. Optineurin was originally identified as a gene responsible for primary open-angle glaucoma [1], a progressive blinding disease, where pathology is due to the loss of the retinal ganglion cells and damage to their axons that make up the optic nerve. Subsequent groups have implicated many different mutations in OPTN with this disease [2]–[20]. Amyotrophic lateral sclerosis (ALS) is a progressive debilitating condition where the loss of spinal motor neurons leads to paralysis and death, and researchers have also found mutations in OPTN to be associated with various forms of this disease [21]–[35]. More recently, mutations in OPTN have been implicated in the metabolic bone disorder Paget’s disease of bone [36]–[38]. Additionally, OPTN immunoreactivity has been found in protein inclusions in a variety of neurodegenerative conditions [39]–[45], although it is not known whether the presence of OPTN in these inclusions is a cause or an effect of the disease pathology. Although OPTN has been associated with all of these diseases, it is still not known how mutations in OPTN contribute to the disease pathology.

Experiments *in vitro* have identified a variety of roles for OPTN and therefore implicate it as a multifunctional adaptor protein. First, OPTN inhibits NF-κB signaling by functioning in the tumor necrosis factor receptor complex [46], [47]. It does this by binding to ubiquitinated RIP (receptor interacting protein) to displace IKBKG (inhibitor of κB kinase gamma), and then bringing in cylindromatosis (CYLD) to deubiquitinate RIP and terminate the signaling pathway [48]–[50]. Second, OPTN is necessary for vesicle trafficking and Golgi maintenance by forming a link between Golgi and transport vesicle membranes by binding RAB8 or membrane-associated receptors, and through interactions with cytoskeletal motor proteins like MYO6 or HTT, which interacts with dynein [1], [51]–[59]. In addition, OPTN has also been shown to aid in RAB8 turnover by forming a complex between RAB8 and its GTPase-activating protein, TBC1 domain family member 17 [60], [61]. Third, OPTN has a role in autophagy by linking the ubiquitinated cargo to microtubule-associated protein 1 light chain 3 (MAP1LC3)-decorated membrane to assemble the autophagosome [62], or through an unknown mechanism [63]. Furthermore, OPTN can couple the MAP1LC3-decorated membrane to myosin VI (MYO6) to aid in trafficking to the lysosome [64]. Fourth, OPTN may have a role in mitosis by translocating into the nucleus and forming a complex between cyclin-dependent kinase 1 and a myosin phosphatase complex (myosin phosphatase targeting subunit 1 and type 1 protein phosphatase catalytic subunit beta) to antagonize polo-like kinase 1 and allow mitotic progression [65]. Finally, OPTN has also been shown to bind metabotropic glutamate receptor 1a (GRM1A) [66] and RAB11A [67], indicating that OPTN may have further roles in vesicle trafficking. The loss of OPTN *in vitro* has been associated with a variety of cellular defects, including an increase in apoptosis [47], [68], [69], increase of NF-κB dependent transcription [47], [48], disruption of Golgi structure [51], [68], defects in vesicle trafficking [51], [54], [58], [70], increase in filopodia [70], defects in directed cell migration [63], [70], disruption of autophagy [62]–[64] and formation of multi-nucleated cells [65], [68]. Collectively, much has been learned about OPTN’s function *in vitro*, but it is unclear how defects in these functions contribute to disease pathology. In addition, the severity of some of these defects, such as cell death, seem to be vary depending on the cell lines used and other experimental methods. The next step to understanding OPTN function is to study it *in vivo*. Here, we can test whether the *in vitro* observations occur with loss of OPTN *in vivo*, and investigate whether potential phenotypes can help explain disease-associated pathology.

There have been a few studies that examined OPTN function *in vivo*, although the results are limited. Two different knock-in mouse lines have been developed that interfere with OPTN function: the OPTN^D477N^ line interferes with ubiquitin binding [71] and the OPTN^470T^ line causes a C-terminal truncation, which would also eliminate the ubiquitin-binding domain [72]. Neither of these papers described any overt phenotype for these mouse lines, with the exception of an increase in embryonic lethality related to the genetic background of the OPTN^470T^ line [72]. There have also been published reports using mouse transgenic lines where OPTN is overexpressed, resulting in progressive retinal degeneration [55], [73]. These lines provide insight into gain of OPTN function, but may not represent an ideal disease model, since OPTN overexpression is not found with disease. In addition, another paper showed that transient knockdown of Optn in zebrafish embryos resulted in abnormal morphology and defects in spinal motor axon guidance, a defect that can be related to problems in directed cell migration [63]. While this work has begun to define OPTN function *in vivo*, a more systematic analysis on the consequences of mutation to the endogenous OPTN locus is needed.

In this paper, we used zebrafish embryos to investigate the cellular and systemic defects of an Optn loss of function mutation. Zebrafish embryos provide an excellent tool to study Optn loss since the optical transparency, *ex vivo* fertilization and multiple transgenic imaging lines allow examination of previously described *in vitro* defects within an *in vivo* context. In addition, as a vertebrate experimental system, zebrafish share a high degree of conservation with mammals for both cell types and molecular signaling pathways, allowing investigation into whether specific cellular defects are relevant to human diseases. Contrary to the previous report using zebrafish, we observed only subtle morphological defects in either genetic mutant or morphant knockdown embryos. We found, however, that loss of Optn resulted in a transient increase in apoptotic cell death throughout the embryos, but this cell death occurred without disruption of the Golgi apparatus. The Optn knockdown embryos did not show acute changes to NF-κB target transcripts or obvious defects in mitosis. We observed transient moderate changes in the cell migration of neural crest cells, but not in other migratory cell types. Finally, we observed changes in the dynamics of vesicle trafficking in spinal axons. We conclude that loss of Optn function in the zebrafish does not phenocopy all of the previously described *in vitro* defects, and future work should focus on apoptosis and vesicle trafficking defects.

## Materials and Methods

### Animals

Zebrafish (*Danio rerio*) were maintained in a laboratory breeding colony on a 14/10 hour light/dark cycle. Embryos were maintained at 28.5°C and staged as described previously [74]. Embryo and fish ages were defined as hours post-fertilization (hpf), days post-fertilization (dpf) or months post-fertilization (mpf). Embryos were obtained through natural mating and placed directly into system fish water. N-phenylthiourea (PTU) was added to the rearing water (0.003%) to inhibit melanin synthesis during embryonic development to facilitate better fluorescent microscopy.

The *optineurin (optn)^sa0143^* line (this study) was generated by *N*-ethyl-*N*-nitrosourea (ENU) mutagenesis and identified by TILLING at the Zebrafish Mutation Project at the Wellcome Trust Sanger Institute [75]. We obtained this line from the Wellcome Trust Sanger Institute and maintained the mutant line on the wild-type ZDR background. The Tg(bact:Man2A(1–100)-EGFP)^mw4^ (this study) was generated using Gateway (Life Technologies, Grand Island, NY) recombination and the Tol2kit [76] with the Man2a(1–100) construct [77]. Generation of the stable line was performed by injection of the circular plasmid with *transposase* RNA as previously described [78], [79]. The Tg(kdrl:EGFP)^s843^ [80] and Tg(-7.2sox10:EGFP)^ir937^ [81] zebrafish lines have previously been described. Wild-type Tu or WIK zebrafish were also used as controls.

### Ethics statement

This study was carried out in strict accordance with the recommendations in the Guide for the Care and Use of Laboratory Animals of the National Institutes of Health. The protocol was approved by the Institutional Animal Care and Use Committee of the Medical College of Wisconsin (protocol number AUA1378). Zebrafish were anesthetized using 3-amino benzoic acid ethylester (tricaine) at 0.02% for embryos and 0.05% for adults, buffered to pH 7.2, and all efforts were made to minimize suffering.

### RNA isolation and generation of cDNA

Embryonic tissue consisted of whole embryos or dissected eyes, which were placed directly into Trizol (Life Technologies). Adult fish were decapitated and the retinas were dissected from the rest of the ocular tissue using Dumont no. 5 forceps and the tissue was placed into Trizol. RNA was extracted using standard protocols and cDNA was reverse-transcribed with SuperScript III First-Strand Synthesis System (Life Technologies).

### Cloning of zebrafish optineurin

The cDNA was generated from eye tissue from 48 hpf Tu embryos and *optn* (Accession number BC063986) was amplified using primers against the coding sequence:

Optineurin cds F: 5′–ATGGCATCTGGATCATCGATGATG–3′
Optineurin cds R1 w stop: TCAGATGATGCAATCCATGATGTG–3′

The product was separated on an agarose gel to confirm a band of the correct size, and gel purified with QIAquick Gel Extraction Kit (Qiagen, Valencia, CA). The purified DNA was amplified further with primers to add attB recombination sites for cloning:

Optineurin cds attB1 F: 5′–GGGGACAAGTTTGTACAAAAAAGCAGGCTATATGGCATCTGGATCGATGATG–3′
Optineurin cds attB2 R w stop: 5′–GGGGACCACTTTGTACAAGAAAGCTGGGTTCAGATGATGCAATCCATGATGTG–3′

The PCR product was then cloned into the pDONR/Zeo plasmid with the Gateway kit (Life Technologies) and transformed into bacteria. The plasmid was recovered with QIAprep Spin Miniprep Kit (Qiagen, Valencia, CA) and analyzed by restriction digest with AlwNI and NcoI (New England Biolabs, Ipswich, MA). The constructs were then sequenced (Davis Sequencing, Davis, CA).

### Sequence analysis

Human (ENSG00000123240) and mouse (ENSMUSG00000026672) OPTN sequences were obtained from the Ensembl database. Sequences were aligned using MegAlign software (DNASTAR, Madison, WI) and the Jotun Hein Method. This alignment was then used to determine the equivalent protein binding sites in the mouse and zebrafish sequences, and the conservation of disease-associated residues.

Sequence similarity of full-length proteins and protein binding sites was determined using NCBI blastp (http://blast.ncbi.nlm.nih.gov/Blast.cgi) using default settings. An E-value threshold of 10E-10 was used to determine significance [82].

Secondary structure prediction was performed using the PSIPRED server (http://bioinf.cs.ucl.ac.uk/psipred/) and DisEMBL tools (http://dis.embl.de/) with default settings.

### *In situ* hybridization and immunohistochemistry

Whole mount *in situ* hybridization was performed as previously described [83] with one modification. Anti-sense RNA probe was purified using a ProbeQuant G-50 micro spin column (GE Healthcare, Pittsburgh, PA). After staining was complete, some embryos were further processed for sectioning. These embryos were infiltrated with sucrose, followed by HistoPrep embedding medium (Fisher Scientific, Pittsburgh, PA) and frozen. Embryos were then sectioned transversely using a Bright OTF5000 cryostat (A-M Systems, Carlsborg, WA).

Immunohistochemistry was performed as previously described [84]. Primary antibodies used were: monoclonal mouse anti-ZN-12 (1∶500; recognizes HNK-1, Zebrafish International Resource Center, Eugene, OR), monoclonal mouse anti-SV2 (1∶50; Developmental Studies Hybridoma Bank, University of Iowa, Iowa City, Iowa), monoclonal mouse anti-acetylated alpha-tubulin (1∶1500, T7451, clone 6-11B-1; Sigma-Aldrich, St. Louis, MO), and polyclonal rabbit anti-cleaved caspase-3 primary antibody (1∶500, 9661; Cell Signaling Technology, Danvers, MA). Labeling with the SV2 antibody required additional washes through methanol and acetone as previously described [85]. Labeling with the caspase-3 antibody required alternative washing and blocking solutions [86]. Antibody labeling was visualized with Alexa 488- (1∶1000, Life Technologies) or Rhodamine Red-X- (1∶750, Jackson Immunoresearch, West Grove, PA) conjugated secondary antibodies.

### Sequencing of *sa0143* mutation

Genomic DNA from caudal fin tissue was digested using the Puregene Core Kit A (Qiagen, Valencia, CA). The DNA flanking the mutation was then amplified using a nested PCR using standard methods, first by primers 1 and 4, and then by primers 2 and 3.

Optn 1: 5′–TGCTTTGAAGTGTTTATGTTACTTC–3′
Optn 2: 5′–CAAGGCTTAAAGTCCACAATC–3′
Optn 3: 5′–ACACTCGCAACTTTCTTTCC–3′
Optn 4: 5′–TTGGTGTACGCATCTTTCAG–3′

The PCR products were purified using ExoI and SAP (New England Biolabs, Ipswich, MA) and sequenced (Retrogen, San Diego, CA). Sequence files were analyzed by SeqMan software (DNASTAR, Madison, WI).

Adult zebrafish were genotyped using the polymerase chain reaction amplification of specific alleles protocol [87]. The R1 primer recognizes the WT allele and R2 primer recognizes the sa0143 allele. Primers for genomic *ef1a* were used as a positive loading control.

Optn sa0143 PASA R1: 5′–GCCCAAATCCTGCTCCA–3′
Optn sa0143 PASA R2: 5′–GCCCAAATCCTGCTCCT–3′
Optn sa0143 PASA F1: 5′–GTATGGAGGTTTGGTGCCTTTCGT–3′
EF1S: 5′–TGGGCACTCTACTTAAGGAC–3′
EF1AS: 5′–TGTGCCAACAGGTGCAGTTC–3′

### Microscopy

Color brightfield images were taken of adult fish by first anesthetizing them in 0.05% tricaine and then pictures were taken with a Nikon E5700 camera on a Nikon SMZ 1500 microscope. Embryos were anesthetized in 0.02% tricaine and mounted in 1% low-melting point agarose in fish water. Images were captured using a Nikon Eclipse 80i microscope and NIS Elements software.

Confocal microscopy was performed using a Nikon Eclipse E600FN or E800 confocal microscope. Images were generated using the Nikon EZ-C1 viewer (Nikon Instruments, Melville, NY), Adobe Photoshop and Illustrator (Adobe Systems, Inc., San Jose, CA), and Metamorph (Molecular Devices, Inc., Sunnyvale, CA) software.

### Western blotting

Embryos were anesthetized with tricaine and mechanically homogenized with a Dounce homogenizer in NP40 buffer (2.5 µl per embryo) at 4°C. The protein was then spun down at 4°C for 20 minutes and the supernatant was aliquoted and frozen for storage at −80°C. Western blotting was performed using standard techniques with 4–20% Tris-glycine gel and transferred to PVDF membrane (all from Bio-Rad, Hercules, CA). Membranes were blocked with Odyssey blocking buffer, incubated in primary and secondary antibodies and imaged using the LI-COR Odyssey infrared imaging system (LI-COR Biosciences, Lincoln, NE). Antibodies used were: polyclonal rabbit anti-Optineurin (C-term) (1∶1000, 100000; Cayman Chemical, Ann Arbor, MI), monoclonal mouse anti-gamma-tubulin (1∶1000, T6557, clone GTU-88; Sigma-Aldrich), IRDye800CW-conjugated goat anti-rabbit IgG and IRDye680-conjugated goat anti-mouse IgG (1∶1500, LI-COR Biosciences, Lincoln, NE). Western blots were performed at least three times with unique biological samples and yielded similar results. Each sample was a pool of 10 embryos from a single clutch.

### Morpholino knockdown

Antisense oligonucleotide morpholinos were designed against the *optn* transcript and obtained from Gene Tools (Corvallis, OR, USA). The morpholinos were prepared and injected as previously described [88]. The control morpholino spans an intron/exon boundary of *slc24a3*, a non-essential gene for embryonic development.

MO3-optn: 5′–TAACCCGCACCTTTCAGGTCTCGGT–3′
MO4-optn: 5′–TGTCCCCATTCATCATCGATGATCC–3′
Ctl MO: 5′–TTACTGACCTCTGTAATGAGCATTC–3′

### Semiquantitative and quantitative reverse transcriptase PCR

The cDNA was generated from pools of embryos (10 embryos per sample) or retinas from 6 mpf adult fish (2 retinas from one fish per sample). Alternative splicing with the MO3-optn was analyzed with two sets of primers against the *optn* transcript. E1 P8 and E3 P10 amplify a region spanning exons 1 through 3 and containing the translational start site in exon 2. E2 P4 and E4 P5 amplify a region spanning exons 2 through 5. Primers for that recognize *ef1a* were used as a loading control. Samples were removed between 24 and 30 PCR cycles and electrophoresed on an agarose gel. The resulting bands were sequenced to determine alternative splicing.

Optineurin E1 P8 F: 5′–CAAGTTGCTGTACACAAGACGCGA–3′
Optineurin E3 P10 R: 5′–TTCTGGCCTCCTCTAATCGTTGCT–3′
Optineurin E2 P4 F: 5′–AGGAGACCCTCCAGCAAATGAACA–3′
Optineurin E4 P5 R: 5′–GTGTGCAAACACTCAGCTCCACTT–3′

*Ef1a* (Gene ID: 30516) primers were previously described [85].

The *optn* transcript levels were analyzed from cDNA generated from pools of 5 dpf WT or maternal and zygotic mutant (*MZoptn*) embryos (10 embryos per sample) or pools of retinae from 3 mpf or 6 mpf WT, *optn* heterozygous or *optn* homozygous fish (2 retinae from single fish per sample). Quantification was performed using the E1 P8 and E3 P10 primers. The experiment was run 3 times with unique biological samples each time. The relative *optn* transcript levels were determined by normalizing to the *ef1a* transcript. Relative amounts of transcript for each experiment were compared to WT levels and the 3 experiments were averaged. Similar results were obtained with the E2 P4 and E4 P5 primers, and by using tissue from other embryonic and adult ages.

Quantitative PCR was performed using the iQ SYBR Green Supermix with the CFX96 Real-Time system (Bio-Rad, Hercules, CA). Samples were run in triplicate and averaged, using *ef1a* as a reference gene. At least three-independent cDNA samples were assayed in triplicate for each primer set. *ef1α* was used for normalization. Data were analyzed using the ΔΔCt method [89]. Primers used were *casp8* (NM_131510.2) [90], *tp53* (30590) [88], *rela* (NM_001001839) [91], *tnfa* (BC165066) [92] and *ikbaa* (AY163840) from [93].

ikbaa F: 5′–CAGCACCTGCGTTCCATTCT–3′
ikbaa R: 5′–GCACGTGTGTCCGCTGTAGT–3′

### Acridine Orange labeling

Live embryos were labeled with Acridine Orange (3,6-bis[dimethylamino]acridine hemi-zinc chloride, Sigma-Aldrich) and imaged as previously described [94].

### Alcian blue labeling

Jaw cartilage was labeled with 0.1% Alcian Blue (Sigma-Aldrich) as previously described [95], except trypsinization was not performed.

### Transmission electron microscopy

Embryos were fixed in 2% paraformaldehyde/2.5% glutaraldehyde and processed as previously described [96]. Transverse sections of the forebrain/midbrain, posterior to the eye, were analyzed from 3 WT and 4 *MZoptn* embryos.

### Dextran trafficking

WT or *MZoptn* embryos at 2 dpf were anesthetized with tricaine and mounted laterally in 1% low-melting point agarose. A small bolus of Texas Red dextran (mol wt. 3000 kD, 6% in PBS, Life Technologies) was injected into the posterior spinal cord and time-lapse images were gathered from an area anterior to the injection site. Only anteriorly trafficking puncta were used for analysis.

### Quantification

The eye area and yolk extension length were measured from images of live embryos at 25 hpf and 5 dpf. Analysis was performed on 8 embryos of each genotype from 2 different clutches.

The isoperimetric quotient of a circular figure is a mathematical formula and is defined as the ratio of (4πA)/L^2^, where A is the area of the figure and L is the outer perimeter. This quotient will equal 1 for a perfect circle and less than 1 for irregular shapes. Using Metamorph software, the threshold was set on images to cover the pigment cells. The software was then used to automatically create regions around each of these objects and calculate the perimeter and area of these regions. Only regions that enveloped whole, isolated cells were used for analysis. Quantifications were performed on 27 cells in 14 embryos from 2 clutches in the WT embryos, and 57 cells in 17 embryos from 2 clutches in the *MZoptn* embryos.

Cell death quantifications with Acridine Orange or caspase-3 labeling were performed similarly. Positively labeled cells were counted from compressed confocal z-stack images. In the heads, cells were counted if they resided anterior to the otic vesicle and dorsal to the yolk. In the trunks, cells were counted if the resided dorsal to the yolk tube. At both 25 hpf and 2 dpf, 19 WT embryos and 20 *MZoptn* embryos were analyzed from 2 different clutches for Acridine Orange labeling. For caspase-3 labeling, 30 WT and 31 *MZoptn* embryos from 2 clutches were analyzed.

For the Golgi labeling, we analyzed z-stacks of the entire trigeminal ganglion from 2 clutches of embryos of at least 10 embryos for each genotype. The HNK-1 staining labeled the plasma membrane of the neurons, allowing us to identify cell boundaries. We tested if the HNK-1 staining and Man2A(1–100)-EGFP were present in a single puncta or multiple fragments within each cell.

Analyses of axon guidance defects at 24 and 48 hpf were performed on at least 2 clutches of 10 embryos each for each genotype and antibody. No defects in axon extension, branching or pathfinding were ever seen. Spinal motor axon length and branching were examined in 26 WT and 24 *MZoptn* embryos from 2 clutches at 25 hpf, and 18 WT and 15 *MZoptn* embryos from 2 clutches at 2 dpf. Additionally, 25 hpf spinal motor axon lengths were measured from 6 neurons per embryo, but no significant difference was found (not shown).

The width of the neural crest streams was measured in 18 WT and 18 *MZoptn* embryos. Lateral images of the GFP labeled neural crest cells were captured in the region dorsal to the yolk tube. The anterior-posterior width was measured for the 6 most anterior streams and these widths were averaged for each embryo.

Analysis of jaw cartilage with transgenic GFP or Alcian blue were carried out on 2 clutches of at least 10 embryos each for each genotype and analysis. No defects in size, shape or position were ever seen.

For the dextran trafficking experiments, the locations of bright puncta were identified in each frame from time-lapse movies. Puncta that did not move during the course of the movie or did not remain associated with the faintly labeled axons were excluded from analysis. The distance traveled by each punctum was measured for each frame. Landmarks on the embryo were used to compensate for drift during imaging. A pause was defined and calculated when a punctum traveled less than 0.5 µm between consecutive frames and ended when the punctum traveled more than that distance between frames. The analysis was performed on 26 puncta from 5 WT embryos and 22 puncta from 6 *MZoptn* embryos.

Graphs were constructed with Microsoft Excel software. All error bars represent the standard error of the mean. Statistical significance was assessed by Student’s *t* test.

## Results

### Protein interaction domains are conserved in zebrafish Optn

**Figure 1:**
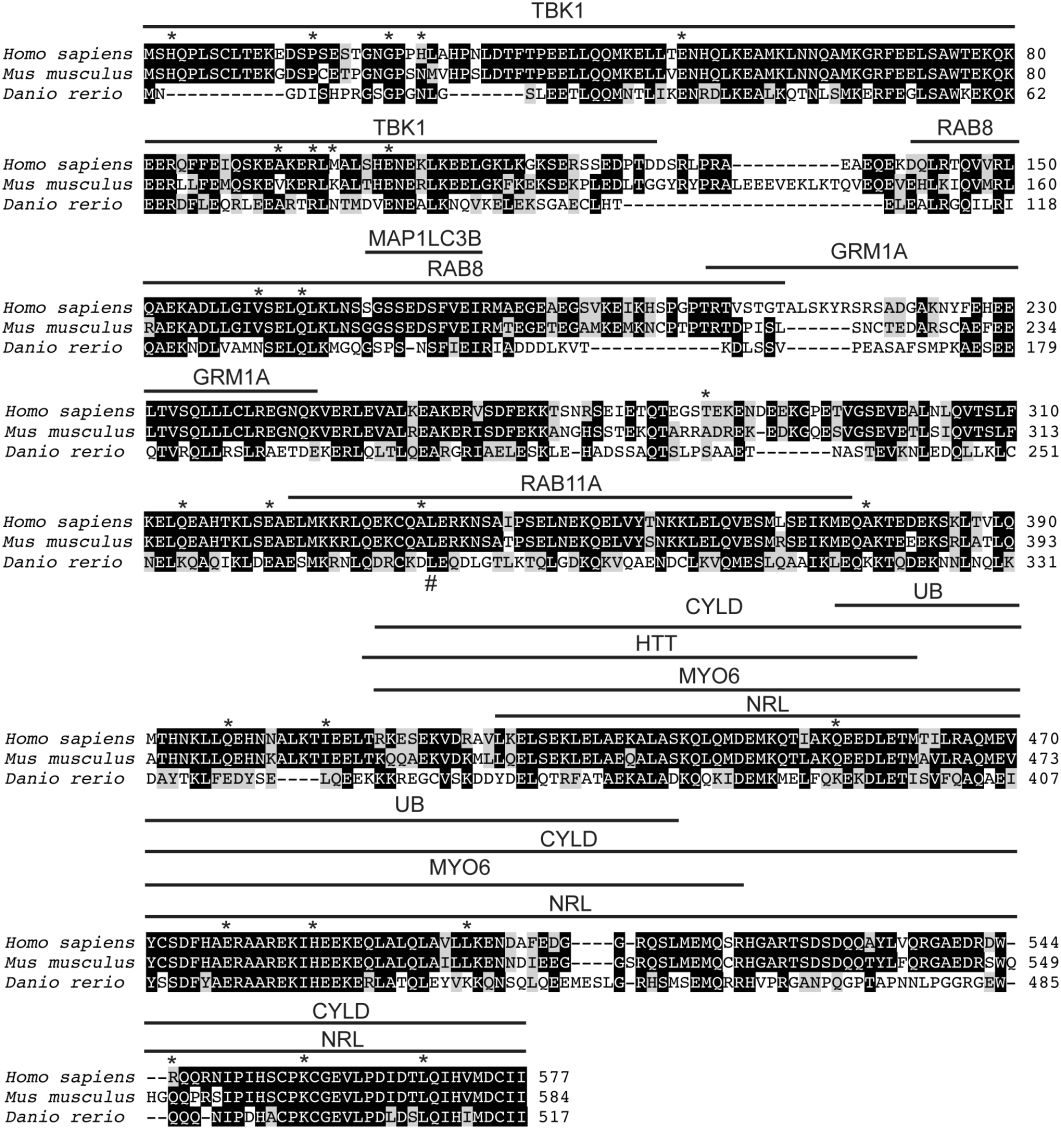
Optineurin protein sequences are conserved across species. Alignment of optineurin protein sequences from human, mouse and zebrafish. Black shaded amino acids are conserved across species, gray shaded amino acids are similar. Identified protein binding sites are indicated above the corresponding parts of the protein. Asterisks indicated amino acids associated with disease in human patients when mutated. Number sign indicates location of *sa0143* zebrafish mutation.

Previously, the zebrafish Optn sequence has been shown to align well to OPTN from humans and other vertebrates [97], but the conservation in regions coding for protein interaction domains has not been characterized. We therefore first isolated zebrafish *optn* from cDNA and confirmed that the nucleotide sequence was identical to published databases. We next compared the protein sequences of OPTN orthologues among human, mouse and zebrafish to examine the overall degree of conservation. We found that full length OPTN was significantly conserved among these different species, similar to the nearest paralogue, IKBKG (Table S1). We also found that the protein sequences for the binding sites for TBK1 [98], MYO6 [51], CYLD [50], UB [49] and NRL [99] were all significantly conserved between human and zebrafish orthologues (visualized in Fig. 1, analyzed in Table S1). This suggests that zebrafish Optn may have similar binding partners and therefore equivalent functions as human OPTN.

Next, we investigated the conservation of residues that have been associated with disease. If a non-synonymous mutation at a specific location is associated with disease, then that residue is likely important for OPTN structure or function. We analyzed each of these disease-associated residues by comparing human OPTN to the orthologous area in mouse and zebrafish proteins. We found that the majority of these residues have equivalent or similar residues in both mouse and zebrafish (asterisks in Fig. 1, Table S2).

Finally, we investigated the predicted secondary structure of the OPTN orthologues. The only published structural analysis was on the MAP1LC3B region of human OPTN, which showed by both X-ray diffraction and by NMR that this region is composed of a β sheet surrounded by α helices [100]. For our analysis, we chose to use the PSIPRED online secondary structure prediction server [101], since this server was able to accurately model the MAP1LC3B binding region. We further used the DisEMBL tool [102] to predict disordered regions which may be important for protein function. The two prediction tools did not completely agree, but overall there was a high level of congruence. Importantly, we found a high degree of similarity between the secondary structures of all three proteins, especially within the binding regions for TBK1, RAB8, MAP1LC3B, MYO6, CYLD, UB and NRL (Fig. S1). Together, this conservation of protein sequence and predicted structure suggests that OPTN function may also be conserved in the zebrafish.

### *Optn* is expressed ubiquitously with enrichment in the head and retina

**Figure 2:**
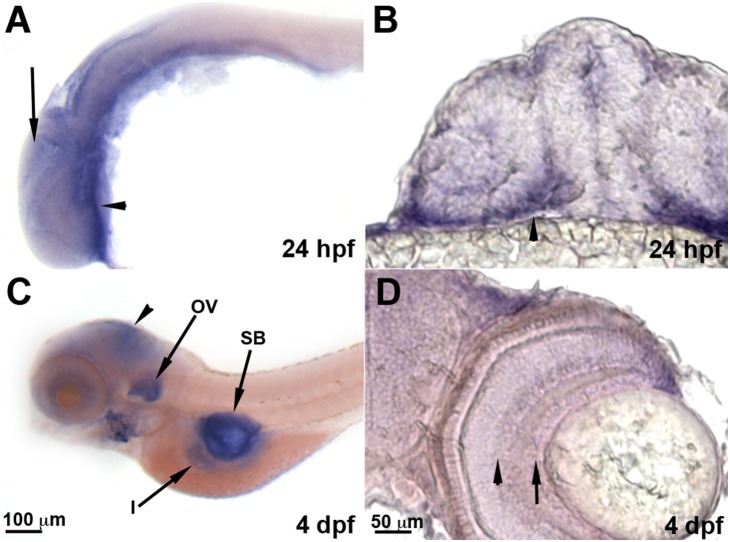
*Optn* is expressed ubiquitously in zebrafish embryos. Whole-mount images of in situ hybridization for *optn* in zebrafish embryos at 24 hpf (A, B) and 4 dpf (C, D). A and C are lateral mounts with anterior to the left and dorsal up. B and D are transverse sections of the head and eyes with dorsal up. **A, B.** *optn* expression is ubiquitous in the 24 hpf embryos (arrow) with some enrichment in the ventral head (arrowheads). **C.** *optn* expression at 4 dpf shows staining in the head (arrowhead) and higher enrichment in the otic vesicle (ov), swim bladder (sb) and intestine (i). **D.** Within the 4 dpf eye, *optn* expression is enriched in the retinal ganglion cell (arrow) and inner nuclear (arrowhead) layers.

We next characterized the expression of *optn* in the zebrafish embryos to determine if certain cell types may be more susceptible to its loss. We found that *optn* was expressed ubiquitously throughout the 1 dpf embryo with enriched labeling in the ventral head (arrowheads in Fig. 2A, B). At 4 dpf, *optn* labeling is enriched in the head (arrowhead), otic vesicle (ov), swim bladder (sb) and intestine (i) (Fig. 2C), although non-specific labeling in the otic vesicle and swim bladder can sometimes occur due to trapping of the reagents in these regions. Within the retina, *optn* labeling is enriched in retinal ganglion (arrow) and inner nuclear (arrowhead) layers (Fig. 2D). This expression pattern is similar to what has previously been described in mouse and human tissues [1], [103], [104].

### *Sa0143* is a null mutation of *optn*

**Figure 3:**
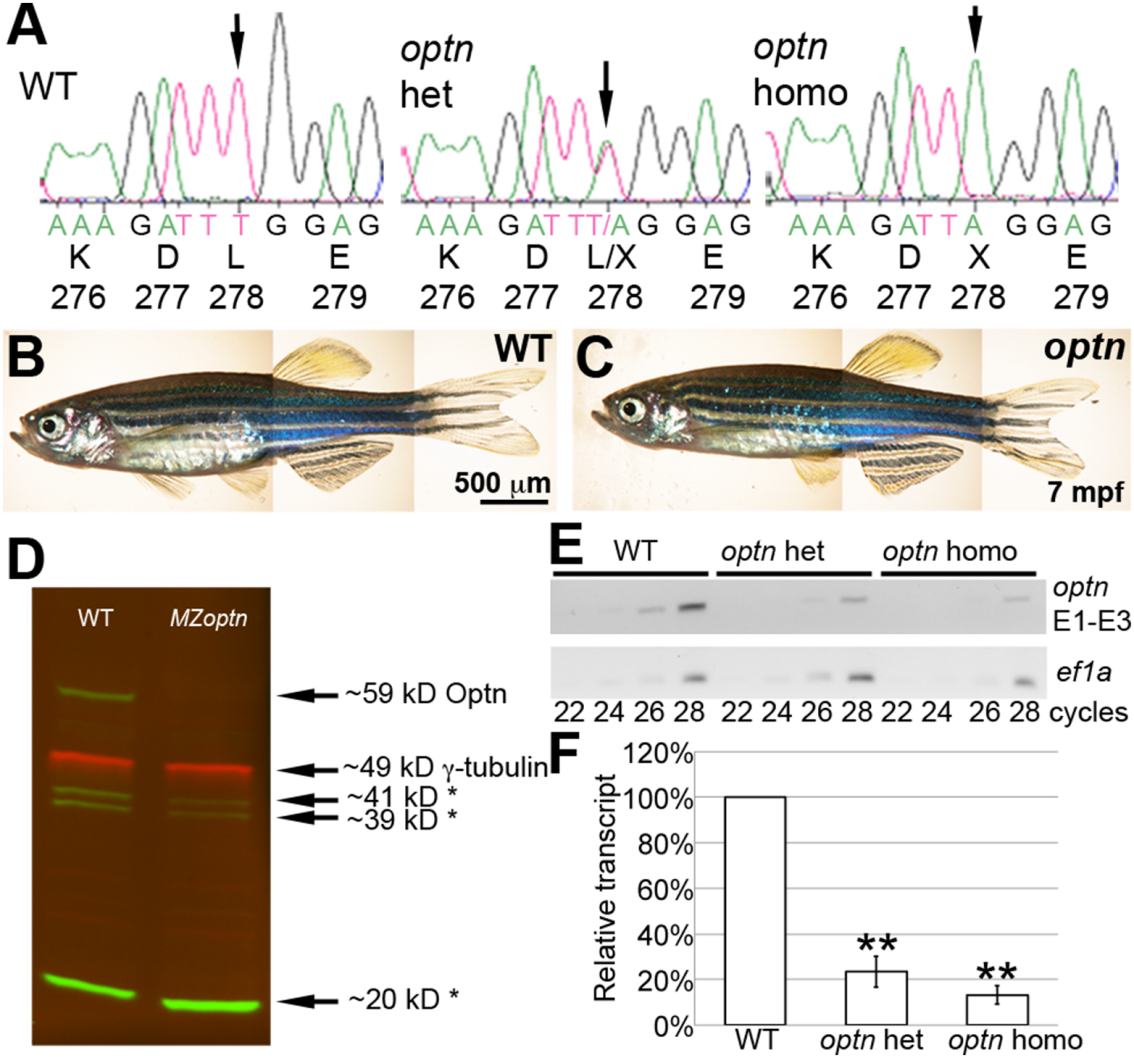
*sa0143* is a null mutation of *optn* with reduced protein and transcript levels. **A.** Trace files from sequencing reactions of adult fish showing region of *sa0143* mutation and corresponding amino acids. Arrow indicates mutated base. **B, C.** Live pictures of adult WT (B) and *optn* (C) fish showing no difference in morphology. **D.** Western blot of protein extracts from 3 dpf embryos. Green = anti-Optn, Red = anti-γ-tubulin as a loading control. Asterisks indicate likely non-specific bands. The Optn band is absent in the *MZoptn* lane. **E.** Representative gel of semi-quantitative RT-PCR run with cDNA from retinas of 6 mpf fish. Top blot was used with primers against exons 1–3 of *optn*, bottom blot was used with primers against *ef1a* as a loading control. PCR was stopped at cycle times indicated below the blots. **F.** Quantification of 3 semi-quantitative RT-PCR experiments. Levels were analyzed for 28-cycle bands and normalized to WT bands for each experiment. ** indicates p≤0.01.

Many mutations in OPTN have been associated with disease, including some that are predicted to eliminate OPTN’s function [1], [4], [21], [22], [24]–[28], [30], [35]. We chose to study the loss of function of Optn in the zebrafish with the *sa0143* (L278X) mutant of *optn* (number sign in Fig. 1) from the Wellcome Trust Sanger Institute [75]. We confirmed the identity of the mutation in the *optn sa0143* mutants from genomic DNA obtained from fin tissue of adult fish. The *optn sa0143* mutation changes a thymine to adenosine, changing the leucine at position 278 to a premature stop codon (Fig. 3A). This mutation is expected to eliminate most or all of the binding sites for CYLD, HTT, MYO6, NRL and UB (Fig. 1), and is therefore predicted to impair Optn function. The adult *optn sa0143* fish did not appear to have any systemic defects, compared to the WT siblings (Fig. 3B, C). The homozygote *optn* mutants displayed no obvious differences in behavior, survivability or fertility over the course of at least 2.5 years of age.

We next confirmed the loss of Optn in this fish line using both adult and embryonic tissue. We incrossed homozygous *optn sa0143* adult parents to generate embryos that are both maternal and zygotic mutants (*MZoptn*) to eliminate any maternal contribution of normal *optn* transcripts or Optn protein that might affect results. The full-length Optn protein was undetected in *MZoptn* embryos compared to WT (Fig. 3D), confirming that this is likely a null mutation. The antibody used for the Western blots recognizes the C-terminus of the protein (amino acids 559–575 of human OPTN), so there remains the possibility that an Optn protein fragment is translated, but is truncated at the premature stop site. Unfortunately, the available antibodies against the N-terminus of human OPTN recognize an area that is absent in the zebrafish protein. Therefore, we also checked mRNA levels to investigate nonsense-mediated mRNA decay of the mutant transcript. RNA from 6 mpf retinae was analyzed by semi-quantitative RT-PCR (Fig. 2E, F). The transcript levels were significantly lower in both the *optn* het (p = 0.007) and *optn* homo (p>0.002) than in WT samples. Similar results were obtained with additional primer sets and with samples from whole WT or *MZoptn* embryos. Together, these results suggest the *optn sa0143* mutants have reduced transcript and undetectable protein levels, similar to that described for the Q398X mutation in human OPTN [30]. The *optn sa0143* mutant zebrafish likely have no functional Optn, and can be used to assess Optn function in the zebrafish.

### *MZoptn* embryos have subtle morphological defects

We next sought to assess potential morphological defects in the *optn sa0143* mutants at various developmental ages. Previous work reported that transient knockdown of Optn in zebrafish embryos resulted in curved tails and a reduced ability to swim [63]. This strong phenotype from Optn knockdown was surprising, as studies on human patients with OPTN mutations have not described any overt defects, besides the progression of the studied disease (ALS, glaucoma or Paget’s disease of bone). However, papers where OPTN was knocked down in various cell culture lines have described numerous defects that should be detrimental to the organism if they occurred widely, including cell death [47], [68], [69], fragmentation of the Golgi [51], [68], and multi-nucleate cells [65], [68]. Therefore, we first sought to assess the general morphology of *optn* mutants at various ages.

**Figure 4:**
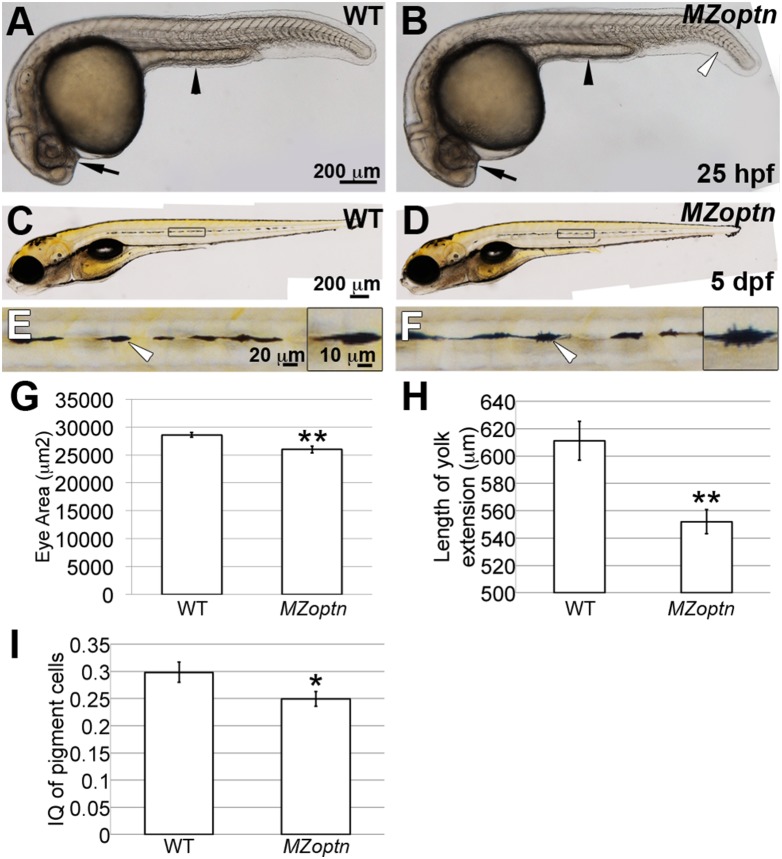
*MZoptn* embryos have subtle morphological defects at early developmental ages. **A-F.** Representative images of live WT (A, C, E) or *MZoptn* (B, D, F) embryos at 25 hpf (A, B) or 5 dpf (C-F). Anterior is to the left. **A, B.** The area of the eyes (arrows) and length of the yolk tube extension (arrowheads) are smaller in *MZoptn* embryos. *MZoptn* embryos occasionally have a curve to the tail (open arrowhead). **C-F.** Pigment cells in *MZoptn* embryos have more filopodia. E and F are higher magnification views of boxed areas in C and D. Insets in E and F are higher magnifications of pigment cells indicated by arrows. **G-I.** Quantification of eye size at 25 hpf (I), length of yolk tube extension at 25 hpf (J) and isoperimetric quotient of pigment cells at 5 dpf (K). * indicates p≤0.05, ** indicates p≤0.01.

Unlike previous observations using morpholinos [63], the majority of *MZoptn* germline embryos appear grossly normal at 25 hpf, although some have a slightly more downwardly curved tail (Fig. 4B, open arrowhead), and none showed defects in muscle contractions. We also found that the area of the eyes of the *MZoptn* embryos were slightly smaller than in the WT embryos (p = 0.003) (Fig. 4A, B, arrows, quantified in Fig. 4G). Furthermore, the yolk tube extension was also shorter in the *MZoptn* embryos than in WT embryos (p = 0.005) (Fig. 4A, B, arrowheads, quantified in Fig. 4H). However, we did not see the severe trunk curvature previously described for transient knockdown of optn [63].

We also generated and tested two different morpholinos to evaluate whether acute knockdown of Optn produces embryonic morphological defects. MO4-optn binds to the translational start site to inhibit translation (Fig. S1A). MO3-optn binds to the boundary of exon 2 and intron 2 to interfere with splicing of the transcript. RT-PCR experiments showed that MO3-optn either excised exon 2 and the start site or included intron 2 to cause a frame shift and early stop codon (Fig. S2A, data not shown). Injection of either morpholino was able to knock down Optn protein at 1 dpf (not shown) and 2 dpf (Fig. S2B). However, Optn morphants do not show overt defects in embryo morphology (Fig. S2C–H).

By 5 dpf, *MZoptn* germline embryos were not morphologically different that the WT embryos in any way we could identify, including no significant differences in eye size or body length (Fig. 4C, D). A closer examination revealed that the pigment cells in the mutant embryos seemed to have more processes than the cells in the WT embryos (Fig. 4 E, F, arrowheads), although they migrated to the correct positions. We quantified this difference in morphology by using the formula for the isoperimetric quotient, providing a measure of circularity on a scale between 0 and 1 (with 1 being a perfect circle) (see Material and Methods). By this test, a cell with more processes will have a greater perimeter distance compared to a similar area, resulting in a less circular object and a lower isoperimetric quotient. Using this measure, the pigment cells in WT embryos had a larger isoperimetric quotient than the cells in *MZoptn* embryos (p = 0.035), suggesting the mutant cells are less circular (Fig. 4I). This phenotype is reminiscent of the increased filopodia in HeLa and A549 cells following OPTN knockdown [70].

### *MZoptn* embryos have increased apoptotic cell death

Previous papers have reported that knocking down OPTN in cell culture lines caused an increase in cell death, although this varied between 7 and 30%, depending on the cell line used and other experimental conditions [47], [68], [69]. The consequences of loss of endogenous OPTN on cell death *in vivo*, however, are unknown. Would cell death be widespread or limited to a few sensitized neuronal cell types that are affected in glaucoma or ALS? We addressed this issue by assessing cell death in *MZoptn* embryos to examine where and when this may be occurring.

**Figure 5:**
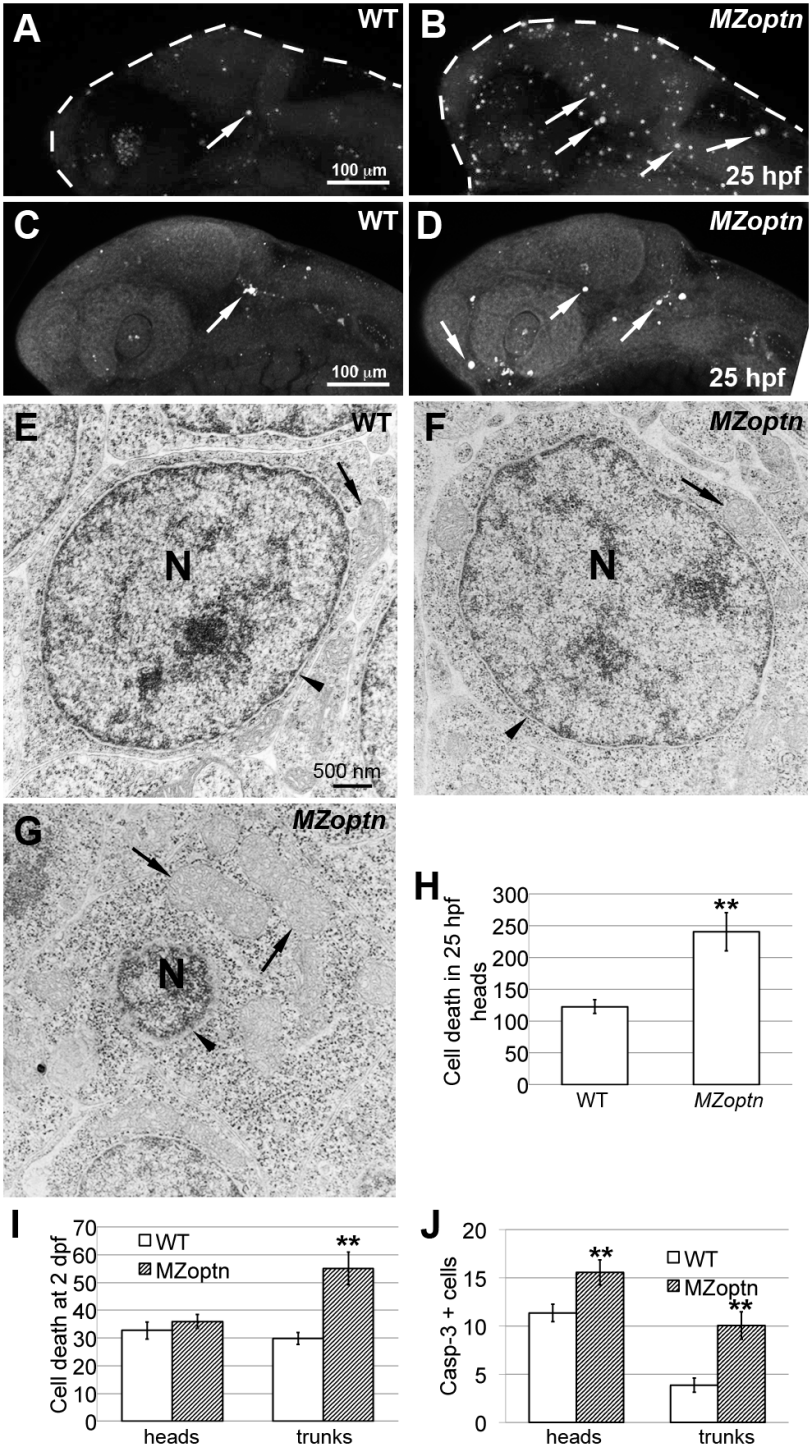
*optn* embryos have increased cell death. **A–D.** Compressed z-stacks of 25 hpf WT (A, C) or *MZoptn* (B, D) embryos labeling dying cells with Acridine Orange (A, B) or α-activated caspase-3 (C, D). Anterior is to the left and dorsal is up. **A, B.** *MZoptn* embryos have more Acridine Orange labeled cells (arrows). **C, D.** *MZoptn* embryos have more caspase-3 labeled cells (arrows). **E–G.** TEM images from cross sections of WT (E) or *MZoptn* embryos (F, G). E, F are normal looking cells, G is apoptotic cell. In G, the nucleus is condensed (N), the nuclear envelope is missing (arrowhead) and the mitochondria are enlarged (arrows) compared to normal cell sin E, F. **H–J.** Quantification of cell death by acridine orange staining in 25 hpf heads (H) or 2 dpf head and trunks (I) or caspase staining in 25 hpf heads and trunks (J). ** indicates p≤0.01.

We saw the greatest morphological defects in *MZoptn* embryos at 25 hpf, and, at this age, we also measured the greatest increase in cell death. The cell death was significantly higher in the *MZoptn* embryos than in the WT embryos (p = 0.001 in the heads) (Fig. 5A, B, arrows, quantified in Fig. 5H). Cell death occurred throughout the embryo, with no clear specificity for organ or tissue types (not shown). However cell death did not seem to increase to levels deleterious to embryonic health since the *MZoptn* embryos still looked fairly normal (Fig. 4A, B) and all survived. By 2 dpf, the cell death in both the WT and *MZoptn* embryos was at much lower levels. We found there was no significant difference in levels of cell death in the heads of these embryos. However, the trunk regions were still significantly different between the WT and the *MZoptn* embryos (p = 0.0005) (quantified in Fig. 5I). By 3 dpf, there were few dying cells in either the WT or mutant embryos, and there was no significant difference between the two groups in any of the regions studied.

There are many different types of cell death that can occur, so we next investigated whether the cell death observed in the *MZoptn* embryos was consistent with apoptosis, as previous papers have reported following OPTN knockdown *in vitro* [68], [69], [105]. We found that the *MZoptn* embryos had higher levels of activated caspase-3 labeling in both the heads (p = 0.01) and trunks (p = 0.0003) at 25 hpf (Fig. 5C, D, arrows, quantified in Fig. 5J), consistent with the cell death being apoptotic. This increase of activated caspase-3 labeling in the heads was similar to the increase in general cell death at 25 hpf (Fig. 5H).

OPTN knockdown has previously been shown to cause apoptosis by upregulating NF-κB signaling by reporter activity or target transcript levels [47], [48]. We tested for this upregulation by performing quantitative PCR for NF-κB target genes (*casp8*, *tp53*, *rela*, *tnfa* and *ikbaa*) *on* 1 dpf embryos. We did not find any significant difference between transcript levels in WT and *MZoptn* embryos (not shown).

We further examined death with ultrastructural analysis. We found that all of the cells examined in the *MZoptn* embryos appeared normal (Fig. 5E, F), with no evidence of multinucleated cells or other abnormalities. Any dying cells we observed in either the WT or *MZoptn* embryos showed hallmarks consistent with apoptosis, including condensed nucleus, loss of the nuclear envelope and enlarged mitochondria (Fig. 5G).

Consistent with cell culture reports, Optn knockdown in the zebrafish does cause an increase in apoptotic cell death. However, this cell death seems to occur transiently during early embryonic ages and does not show any preference for specific tissue types or structures. Although we did see an increase in cell death, it only coincided with subtle morphological differences in the mutant embryos, and these fish appeared morphologically indistinguishable from WT fish at later embryonic and adult ages.

### *MZoptn* embryos do not have Golgi defects

Some papers have described Golgi fragmentation in association with cell death following OPTN knockdown *in vitro* [51], [68]. Similarly, both E478G and Q398X ALS patients had Golgi fragmentation in the anterior horn of the spinal cord in the same area as the dying motor neurons [23], [106]. In both of these cases, it is unclear if the Golgi fragmentation is leading to the cell death or vice versa. Therefore, we next chose to investigate Golgi morphology in the *MZoptn* embryos. Since the *MZoptn* embryos seem overtly normal and can survive into adulthood, it would be surprising if they had widespread Golgi fragmentation. On the other hand, these mutant embryos also have a transiently increased level of cell death, so they may also have other transient deleterious effects at this same time.

**Figure 6:**
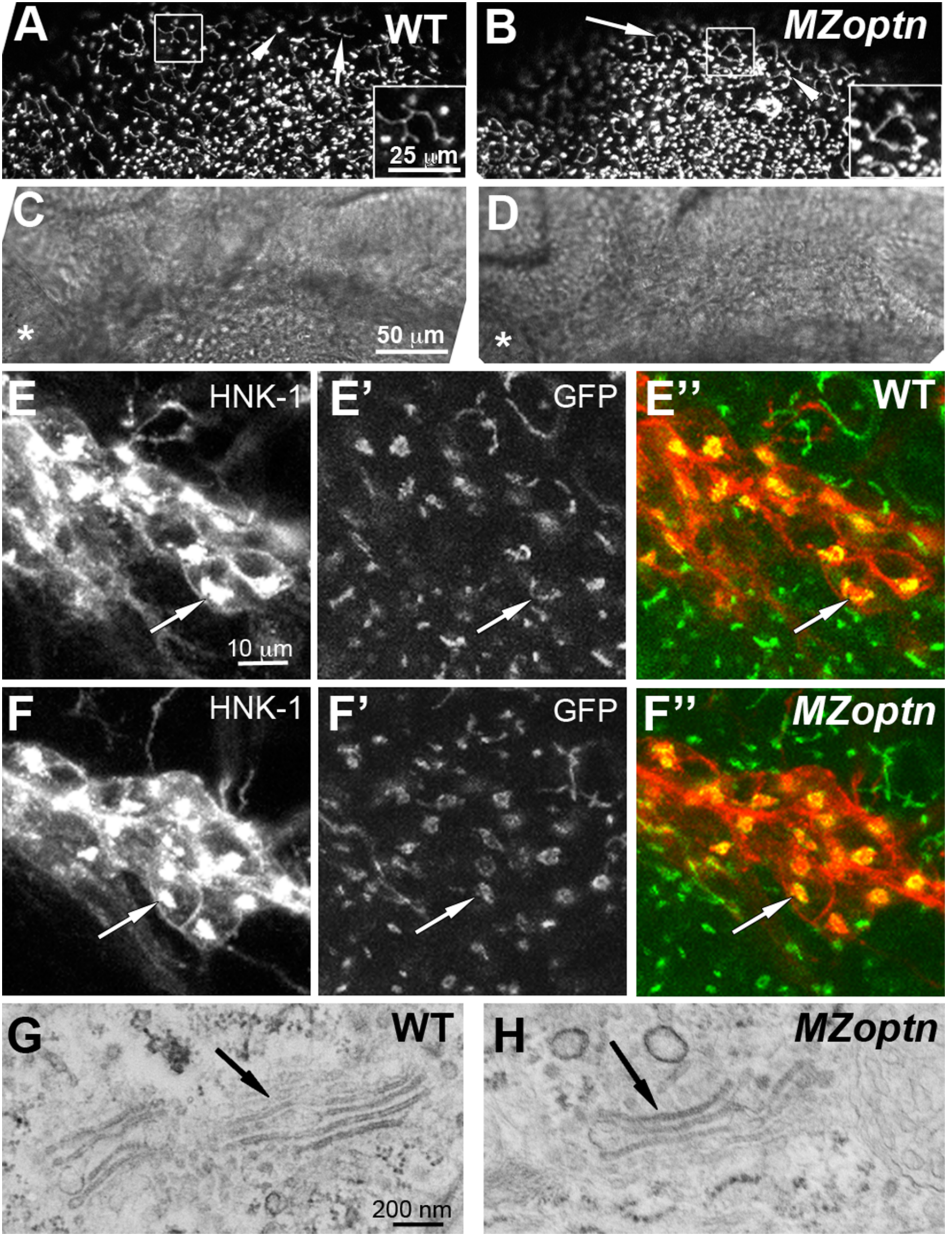
Golgi structure and axon guidance are normal in *optn* embryos. **A–F.** Compressed z-stacks of 25 hpf WT (A, C, E) or *MZoptn* (B, D, F) embryos labeled with man2a-EGFP (A, B, E’, E”, F’, F”) or α-HNK-1 (E, F) or brightfield images (C, D). Insets in A, B are higher magnification views of the boxed regions. C, D are brightfield images of the same regions shown in A, B. Anterior is to the left and dorsal is up. **A–D.** Man2a-GFP labeled Golgi are normal in the midbrain region of *MZoptn* embryos as seen in both the filamentous Golgi of epithelial cells (arrows) and compact Golgi of neuroepithelial cells (arrowheads). Asterisk indicates location of the eye. **E, F.** Co-labeling of the sensory trigeminal nucleus with HNK-1 (E, F), man2a-GFP (E’, F’) or the merge of the two channels (E”, F”). The Golgi looks normal in *MZoptn* embryos (arrows). **G, H.** TEM images of the Golgi in epithelial cells of the head in 25 hpf embryos shows no difference in structure between WT (G) and *MZoptn* (H) embryos (arrows).

We studied the Golgi morphology in the *MZoptn* embryos by crossing the *optn* fish to the Tg(*bact*:Man2A(1–100)-EGFP)^mw4^ line, which expresses a transgenic protein that labels the Golgi with GFP [77]. We imaged the embryos at 25 hpf because this is the developmental age when the cell death is increased in the *MZoptn* mutants. In WT embryos, the superficial epidermal cells had a filamentous Golgi that formed circular structures around the nuclei (Fig. 6A, arrow). The deeper neuroepithelial cells had a more compact Golgi structure (Fig. 6A, arrowhead). We saw no difference in the Golgi structure in either of these cell types in the *MZoptn* embryos (Fig. 6B, arrow and arrowhead). We also did not see any defects in Golgi structure in the Optn morphants (not shown).

We further investigated the Golgi morphology in the sensory trigeminal ganglion cells by co-labeling with an antibody against HNK-1, which labels the Golgi and cell surface of sensory neurons [107]. In both the WT and *MZoptn* embryos, the HNK-1 staining and GFP co-labeled one discrete puncta within the cell (Fig. 6E, F, arrows). The Golgi labeling was never fragmented or scattered throughout the cell, like that seen when OPTN was knocked down *in vitro* [51], [68].

Finally, we examined the ultrastructure of the Golgi in WT and *MZoptn* embryos. We did not identify any abnormal Golgi morphology in any of the embryos (Fig. 6G, H, arrows). All of these data suggest the defects in Golgi structure found in some cell culture lines following OPTN knockdown may be a result of culture conditions or specific cell lines, since they were not seen *in vivo*. However, we cannot rule out species-specific differences in function.

### *MZoptn* embryos have subtle defects in neural crest migration, but other migratory cells are unaffected

OPTN knockdown has previously been shown to cause defects in directed cell migration [70] and cause pathfinding errors in zebrafish spinal motor axons [63], although OPTN had no effect on neurite number or length of neurites in PC12 cells [108]. We investigated a number of migratory cell types in the *MZoptn* embryos to determine if they had defects in their directed migration.

**Figure 7:**
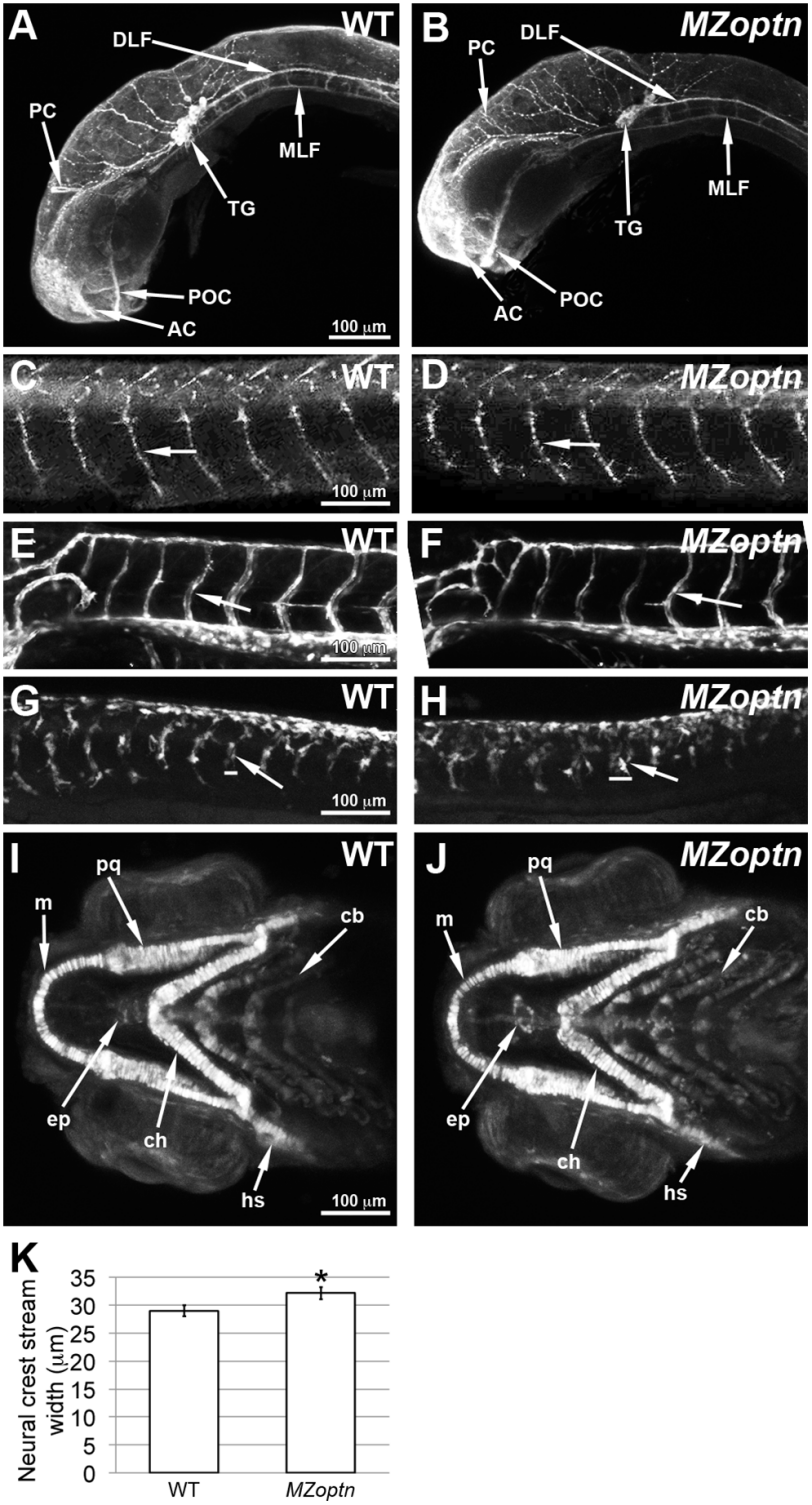
*MZoptn* embryos have defects in the migration of neural crest cells. **A–J.** Compressed z-stacks of WT (A, C, E, G, I) or *MZoptn* (B, D, F, H, J) embryos. Embryos are mounted laterally (A–H) or ventrally (I, J) with anterior to the left. **A, B.** α-acetylated alpha-tubulin staining of axon tracts in 25 hpf embryos. The *MZoptn* embryos have normal axon pathfinding (arrows). **C, D.** α-SV2 staining of spinal motor axons in 2 dpf embryos. These axons are guided normally in *MZoptn* embryos (arrows). **E, F.** GFP labeled endothelial cells in 2 dpf embryos show that intersegmental vessels are patterned correctly in *MZoptn* embryos (arrows). **G, H.** GFP labeling of neural crest cells in 23 hpf embryos. The ventrally directed streams of cells (arrows) in the trunk are in the right location, but the width of the streams is wider in *MZoptn* embryos along the anterior-posterior axis (horizontal bars correspond to width of above stream). **I, J.** GFP labeling of neural crest cells that are contributing to the jaw cartilage in 4 dpf embryos. The elements of the jaw cartilage in *MZoptn* embryos appear normal (arrows), including Meckel’s cartilage (m), palatoquadrate (pq), ceratohyal (ch), hyosympathetic (hs), ethmoid plate (ep) and ceratobranchials (cb). **K.** Quantification of the width of the neural crest streams in G, H. * indicates p≤0.05.

We first examined the axons to determine if there were any defects in the guidance of their growth cones. We labeled the axons of 24 and 48 hpf embryos with antibodies against acetylated alpha tubulin, and HNK-1. We found that all the major axon tracts at these ages appeared normal with no observable defects in axon extension, branching or pathfinding of the anterior commissure (AC), post-optic commissure (POC), posterior commissure (PC), dorsal longitudinal fasciculus (DLF) and medial longitudinal fasciculus (MLF) (Fig. 7A, B). We also saw no differences in the axons of the sensory trigeminal ganglion (TG) (Fig. 7A, B), Rohon-Beard neurons, ephiphysis, and lateral line (not shown). We also labeled the spinal motor axons with the SV2 antibody, similar to the previously reported analysis on zebrafish morphants [63]. Unlike that work, we did not see any defects in either the length or branching of the spinal motor axons at either 24 or 48 hpf (Fig. 7C, D, arrows), and also did not see any defects in muscle contraction that was also described. Like the *MZoptn* embryos, we did not observe any defects in axon guidance or muscle contractions following morpholino knockdown (not shown).

We next investigated the migration of the endothelial cells that make up the blood vessels. We marked these cells by crossing the *optn* fish to the Tg(kdrl:EGFP)^s843^ line [80], and imaged the GFP-labeled endothelia in WT and *MZoptn* embryos at 1 and 2 dpf. We found some occasional errors in the pathfinding of the intersegmental vessels of the trunk in both WT and *MZoptn* embryos, including additional branches or vessels that did not connect properly, but the incidence was not significantly different between the two groups. However, most of the vessels did not have any errors (Fig. 7E, F). Optn morphants similarly had some intersegmental vessel pathfinding errors but these errors were not significantly more frequent than in control embryos (not shown).

Lastly, we examined the migration of neural crest cells by crossing the *optn* fish to the Tg(-7.2sox10:EGFP)^ir937^ line [81]. We imaged the GFP in the neural crest cells in the trunk at 23 hpf. At this developmental stage, the neural crest cells can be seen migrating from the dorsal spinal cord to the ventral trunk in distinct streams. The streams were significantly wider in the *MZoptn* than WT embryos (p = 0.04) (Fig. 7G, H, arrows, quantified in Fig. 7K), but the cells still seemed to localize within the somite boundaries and did not mix together with the neighboring streams. We also investigated neural crest-derived structures (pigment cells and jaw cartilage) to determine if the neural crest cells were able to migrate to their targets in the *MZoptn* embryos. As shown previously, the pigment cells in 5 dpf *MZoptn* embryos correctly reside along the horizontal midline, even though they have increased filopodia (Fig. 4 C–F). The elements of the jaw cartilage had no obvious defects in size, shape and position at 4 dpf, as labeled with the transgenic GFP (Fig. 7I, J), or at 5 dpf, as labeled with Alcian Blue (not shown).

### *MZoptn* embryos have defects in vesicle trafficking

OPTN knockdown has also been associated with defects in vesicle trafficking [51], [54], [58], and these defects have been associated with increased filopodia and defects in directed cell migration [70], two phenotypes that we have found in the *MZoptn* embryos. We investigated vesicle trafficking in the *MZoptn* embryos by injecting Texas-Red labeled dextran into the posterior spinal cord and imaging the trafficking of the fluorescent puncta as they travel anteriorly through the longitudinal axons. The dextran has previously been shown to be endocytosed and retrogradely trafficked through axons [109]. We hypothesized it would be easier to measure small changes in vesicle trafficking in the axons, since the vesicles had to move along a relatively larger distance compared to more compact cells.

**Figure 8:**
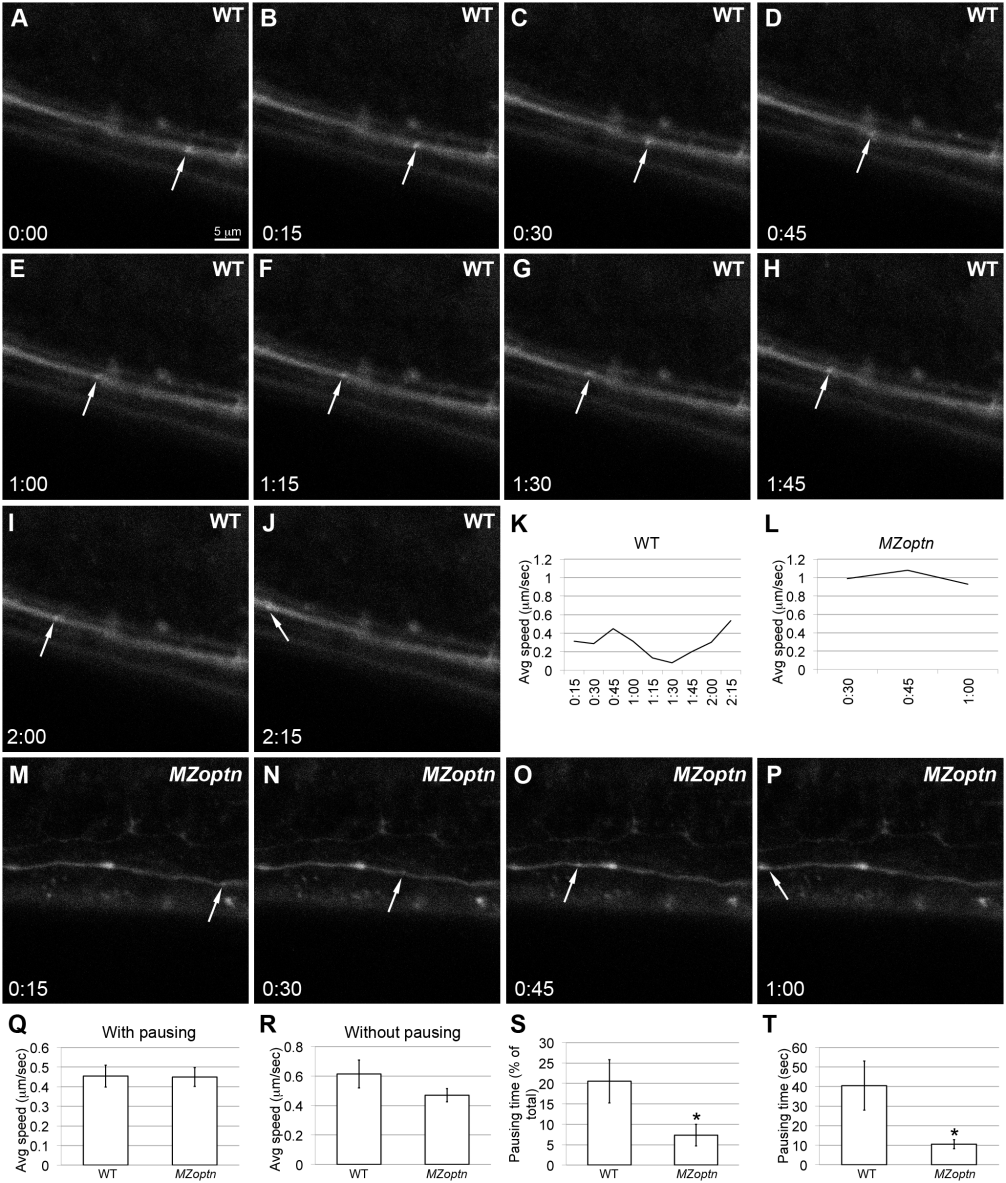
*MZoptn* embryos have defects in the dynamics of vesicle trafficking. **A–J, M–P.** Frames from time-lapse imaging of Texas Red-dextran trafficked through longitudinal spinal axons in WT (A–H) or *MZoptn* (M–P) embryos. Moving puncta are indicated by arrows and time codes are indicated. WT punctum shown in A–K traveled a little slower than average. *MZoptn* punctum shown in L–P traveled faster than average. **K, L.** Quantification of the speed of indicated puncta of WT panels (K) or *MZoptn* panels (L). Each time-point corresponds to the average speed of the puncta over the preceding 15 seconds. **Q–T.** Quantification of all puncta from WT and *MZoptn* embryos showing overall speed of puncta (Q), speed of puncta when they are not pausing (R), % of time puncta spend pausing (S) and time that each puncta spends on individual pauses (T). * indicates p≤0.05.

We found that individual puncta in the WT embryos traveled in a saltatory manner. There were times when they traveled faster (Fig. 8A–E, H–J), and other times when they traveled slower, or spent considerable time pausing (Fig. 8E–H, illustrated in Fig. 8K). Puncta in *MZoptn* embryos tended to go at a more consistent speed, with less time slowing down and pausing (Fig. 8M–P, illustrated in Fig. 8L). Overall, we found that puncta in the WT embryos traveled at the same average speed as in the *MZoptn* embryos (p = 0.94) (Fig. 8Q). When we factored only the times when the puncta were moving and not pausing, then the puncta in the WT embryos tended to travel faster than the ones in the *MZoptn* embryos, but not to a significant level (p = 0.18) (Fig. 8R). We did see a significant reduction in the *MZoptn* embryos for the percentage of the total time the puncta spent paused (p = 0.03) (Fig. 8S) and the average time for each individual pause (p = 0.02) (Fig. 8T). In summary, axonal puncta in *MZoptn* embryos travel moderately slower and at a more constant velocity than in WT embryos.

## Conclusions

In this study, we examined *MZoptn* zebrafish embryos for defects that have been described for OPTN knockdown *in vitro*. First, we found that the *MZoptn* embryos have smaller eyes and a shorter yolk tube extension at an early developmental age, but these defects recover at later ages. Second, we also found that *MZoptn* embryos have increased apoptotic cell death at early ages, but this cell death does not correspond to defects in Golgi morphology or an increase in pro-apoptotic or NF-κB target transcripts. Third, the neural crest cells in the trunks of *MZoptn* embryos take a wider anterior-posterior path during their migration. This does not appear to affect their migration to their targets though, since both the pigment cells and jaw cartilage were present in their normal positions. The pigment cells did have an increase in filopodia, however. Other migratory cell types, such as endothelial cells and the growth cones of axons did not have any significant defects in position. Finally, we determined that the dynamics of vesicle trafficking were altered in the *MZoptn* embryos, with trafficked puncta moving slower, but spending less time pausing. Overall, we saw some defects that were similar to those described from *in vitro* experiments, but not others.

None of our models of Optn loss in the zebrafish yielded the dramatic phenotypes as the previous paper reported [63]. In that study, morphant embryos had a severely curved body, and defects in spinal motor axon guidance and muscle contraction, but we did not see anything that severe. We showed that the *MZoptn* embryos and morphants in this paper all had loss of Optn protein, so this discrepancy is not a matter of read-through of the mutation or inefficient knockdown by the morpholinos. Some morpholinos can have off-target effects due to binding additional targets or general toxicity [110]. For our studies, we used morpholinos that bound to different sequences in the *optn* transcript, so this may help explain our conflicting results. There may also be a difference in genetic background that makes some zebrafish more sensitive or insensitive to Optn loss.

We saw increased cell death in the *MZoptn* embryos, but it did not appear to be as high as what was previously described *in vitro* [47], [68], [69]. The cell death in the *MZoptn* embryos also did not seem to be localized to any specific structure or cell type, so it is unknown why some cells died while others were spared. It is possible that cells are sensitized toward apoptosis by the loss of Optn, but an additional insult is needed to push them towards death.

The increased cell death in *MZoptn* embryos could be the result of other defects associated with OPTN knockdown, such as loss of Golgi structure, defects in mitosis causing multinucleated cells or increased NF-κB signaling. It is possible that only a minority of sensitized cells had Golgi defects or increases in NF-κB signaling, but were below our ability to detect them. NF-κB signaling has previously been investigated in zebrafish embryos with morpholino knockdown of IκB kinase. These embryos had upregulated expression of NF-κB target genes and defects in their tails and brains [93]. *MZoptn* embryos do not resemble the IκB morphants, suggesting any alterations in NF-κB signaling in the *MZoptn* embryos must not be very severe or widespread. Motor neurons with fragmented Golgi have also been described for an ALS patient with a heterozygous E478G mutation in *OPTN* [23]. This may suggest that the progression of Golgi fragmentation with OPTN loss may require additional insults or mutations, or that Golgi fragmentation is specific to OPTN loss in human cells. We can conclude that defects in Golgi structure, cell division or NF-κB signaling are not a general consequence of Optn loss.

The vesicle trafficking defects measured in *MZoptn* embryos were interesting because the dynamics of trafficking were affected while the overall rate of trafficking was not. This phenotype is particularly intriguing because defects in axonal trafficking have been hypothesized to lead to pathology in glaucoma [111], [112] and ALS [113]–[117]. Since retinal ganglion and spinal motor axons are especially long, with high energy requirements, they may be especially susceptible to even relatively small defects in trafficking. These defects may also lead to subtle, but chronic damage that can accumulate over time, sensitizing these cells to pathology later in life. It is possible that trafficking of larger organelles, like mitochondria, could be more severely affected.

The increase in cell death seen with loss of OPTN is often discussed in relation to the neuronal cell death that is seen in patients with glaucoma or ALS. However, it is important to consider that these patients do not have widespread systemic defects that would be consistent with a high rate of cell death. Instead, individuals appear fine until at least middle age when a very specific cell population begins dying (retinal ganglion cells in glaucoma and spinal motor neurons in ALS). Therefore, certain cell types are likely sensitized to cell death by OPTN loss and this death is probably reliant on other genetic and environmental factors.

The experiments in this paper were conducted to address the cellular defects that occur with Optn knockdown, and so were limited to the embryonic ages when these experiments are easiest to perform. We wanted to know whether the *in vitro* defects could be generally applied to any cell type, and for many of these defects the answer was no. However, the ultimate goal of studying OPTN is to better understand how it contributes to diseases, such as glaucoma and ALS. To address this question, we plan to continue our studies of the *optn* fish into adult ages and determine whether they develop pathology similar to glaucoma or ALS.

In conclusion, we hypothesize that Optn does not play an essential role for all of the cell biological processes implicated from *in vitro* studies. The embryonic loss of Optn phenotypes are moderate and predominantly transient. With increased age or in combination with unknown risk factors, the subtle defects may accumulate damage over time, ultimately contributing to disease.

## Supporting Information

Figure S1 **Optineurin secondary structure is conserved across species.** Alignment of secondary structure of optineurin protein from human, mouse and zebrafish using the protein sequence alignment from Figure 1. Open boxes represent predicted α helices, closed boxes and arrowheads represent predicted β sheets and connecting lines represent coiled regions. Open regions represent gaps in the protein sequence alignment from Figure 1. Red colored regions represent predicted disordered regions. Identified protein binding sites are indicated above the corresponding parts of the protein.
(TIF)

Figure S2 **Morpholino knockdown does not affect embryo morphology. A.** Schematic diagram of morpholino binding and alternate splice products. T1 shows the normal unprocessed *optn* transcript. The start codon (S) is in exon 2. Boxed areas denote exons (E1–E4) and horizontal lines denote introns. MO4-optn binds to the start site to inhibit translation initiation. MO3-optn binds to the exon 2/intron 2 boundary to inhibit proper splicing. T2 shows the mature *optn* transcript after proper splicing in control embryos. T3 shows one alternative transcript from MO3-optn binding. Exon 2 has been excised, removing the start site. T4 shows the other alternative transcript from MO3-optn binding. Intron 2 remains, causing a frame shift and early stop codon (asterisk). **B.** Western blot from 2 dpf morpholino injected embryos. The labels above the lanes (C–H) refer to the same morpholino type and concentration as pictured in panels C–H. The green Optn band is present in the ctl MO lanes, but absent in the MO3-optn or MO4-optn lanes. Other green bands (NS) are non-specific bands recognized by the Optn antibody. Red bands are γ-tubulin, used as a loading control. **C–H.** Live pictures of 26 hpf embryos injected with ctl MO (C, D), MO3-optn (E, F) or MO4-optn (G, H) at concentrations of 100 µm (C, E, G) or 200 µm (D, F, H). Anterior is to the left and dorsal is up.
(TIF)

Table S1 **Protein binding sites of OPTN are conserved across species.** The sequences for full-length OPTN or identified protein binding sequences on the OPTN protein were blasted in either mouse or zebrafish protein databases and the likely orthologue was determined. The percent identity between the protein or partial sequences and the corresponding E-value are listed. For comparison, the analysis for the full-length sequence of IKBKG, the nearest paralogue to OPTN, is given.
(DOCX)

Table S2 **Disease-associated residues in optineurin are conserved across species.** Non-synonymous amino acid substitutions in OPTN were analyzed. For each mutation, the corresponding SNP designation, if applicable, and the amount of conservation of the similar region in mouse and zebrafish sequences are listed. Also listed are the disease association and reference for each mutation. Y = conservation of amino acid, N = no conservation, * = species has disease-associated residue.
(DOCX)

Movie S1 **WT vesicles make frequent pauses.** Texas-Red dextran being trafficked through longitudinal spinal axons in WT embryo. Anterior is to the left and dorsal is up. Field of view is 50 µm×50 µm. Images were captured every 5 seconds. Total time shown is 19 minutes, 55 seconds.
(MP4)

Movie S2 ***MZoptn*** **vesicles travel at a more consistent rate with fewer pauses.** Texas-Red dextran being trafficked through longitudinal spinal axons in *MZoptn* embryo. Anterior is to the left and dorsal is up. Field of view is 50 µm×50 µm. Images were captured every 5 seconds. Total time shown is 19 minutes, 55 seconds. Images were captured every 5 seconds. Total time shown is 19 minutes, 55 seconds.
(MP4)
