# Supplementary figures and images for: Loss of Optineurin In Vivo Results in Elevated Cell Death and Alters Axonal Trafficking Dynamics
Source: PLoS One. 2014 Oct 16;9(10):e109922. doi: 10.1371/journal.pone.0109922 (PMC4199637; doi:10.1371/journal.pone.0109922)

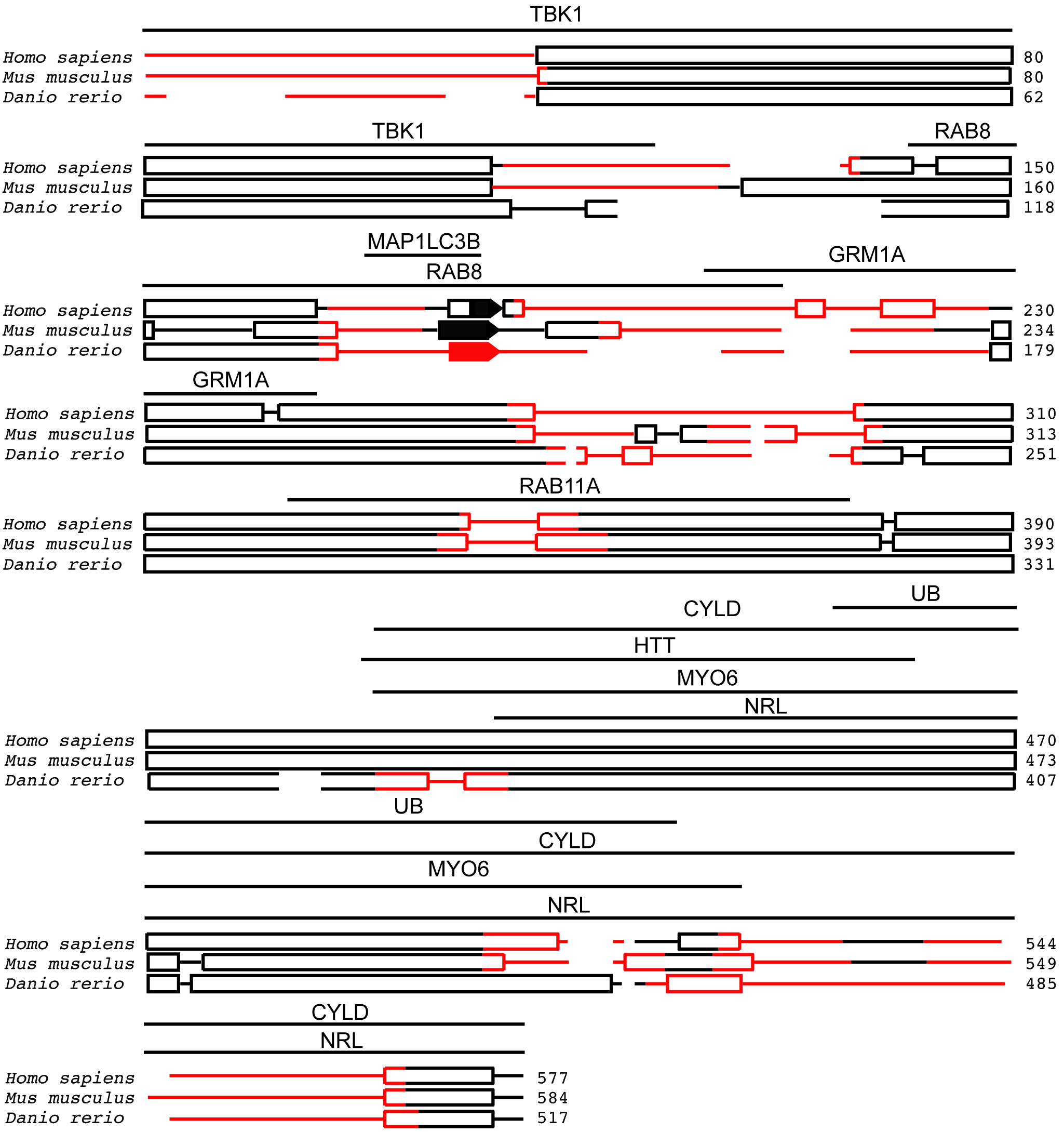

Supplement: Figure S1 — Optineurin secondary structure is conserved across species. Alignment of secondary structure of optineurin protein from human, mouse and zebrafish using the protein sequence alignment from Figure 1. Open boxes represent predicted α helices, closed boxes and arrowheads represent predicted β sheets and connecting lines represent coiled regions. Open regions represent gaps in the protein sequence alignment from Figure 1. Red colored regions represent predicted disordered regions. Identified protein binding sites are indicated above the corresponding parts of the protein. (TIF) [file pone.0109922.s001.tif]

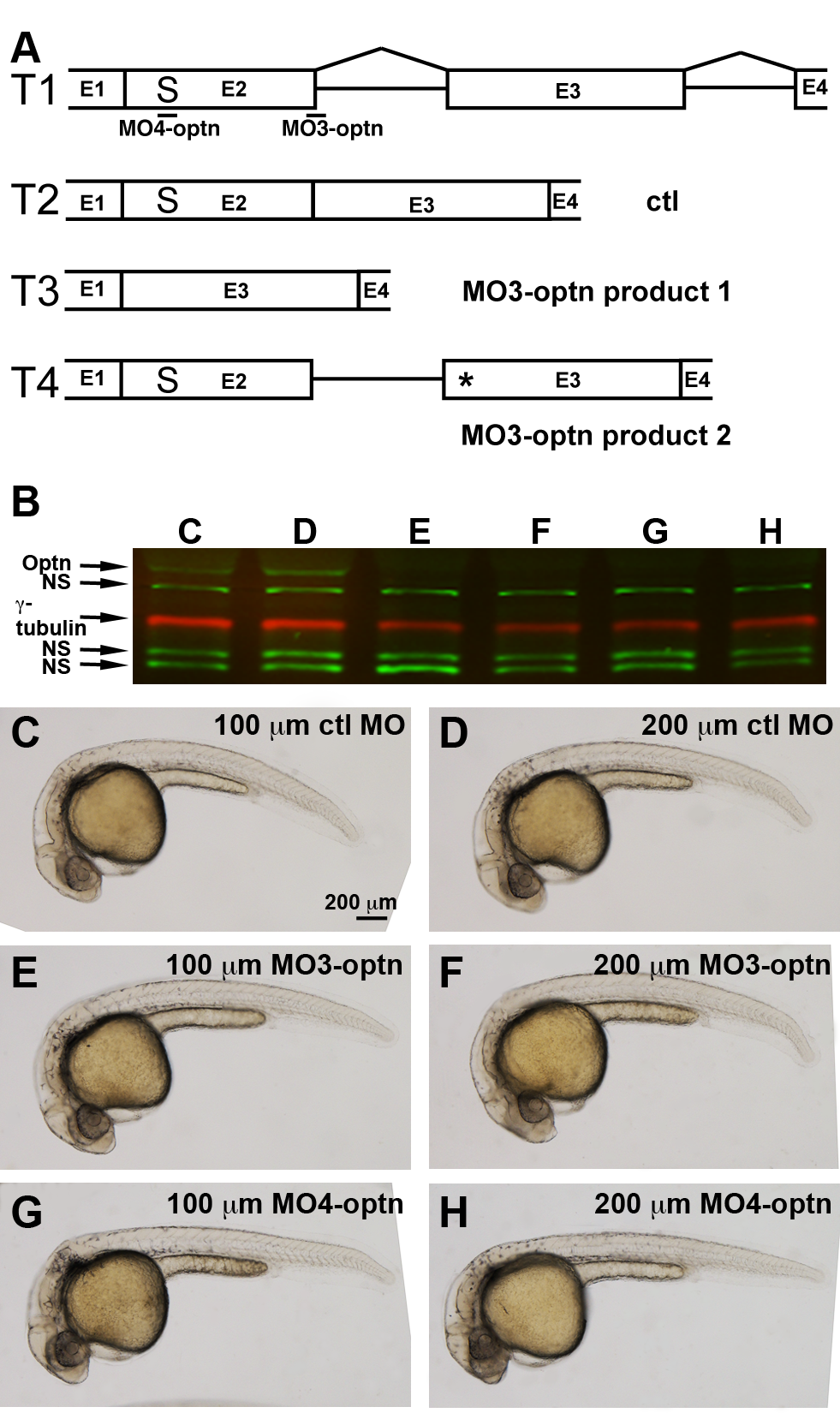

Supplement: Figure S2 — Morpholino knockdown does not affect embryo morphology. A. Schematic diagram of morpholino binding and alternate splice products. T1 shows the normal unprocessed optn transcript. The start codon (S) is in exon 2. Boxed areas denote exons (E1–E4) and horizontal lines denote introns. MO4-optn binds to the start site to inhibit translation initiation. MO3-optn binds to the exon 2/intron 2 boundary to inhibit proper splicing. T2 shows the mature optn transcript after proper splicing in control embryos. T3 shows one alternative transcript from MO3-optn binding. Exon 2 has been excised, removing the start site. T4 shows the other alternative transcript from MO3-optn binding. Intron 2 remains, causing a frame shift and early stop codon (asterisk). B. Western blot from 2 dpf morpholino injected embryos. The labels above the lanes (C–H) refer to the same morpholino type and concentration as pictured in panels C–H. The green Optn band is present in the ctl MO lanes, but absent in the MO3-optn or MO4-optn lanes. Other green bands (NS) are non-specific bands recognized by the Optn antibody. Red bands are γ-tubulin, used as a loading control. C–H. Live pictures of 26 hpf embryos injected with ctl MO (C, D), MO3-optn (E, F) or MO4-optn (G, H) at concentrations of 100 µm (C, E, G) or 200 µm (D, F, H). Anterior is to the left and dorsal is up. (TIF) [file pone.0109922.s002.tif]
