# Supplementary material for: Loss of Optineurin In Vivo Results in Elevated Cell Death and Alters Axonal Trafficking Dynamics
Source: PLoS One. 2014 Oct 16;9(10):e109922. doi: 10.1371/journal.pone.0109922 (PMC4199637; doi:10.1371/journal.pone.0109922)
Supplement: Table S1 — Protein binding sites of OPTN are conserved across species. The sequences for full-length OPTN or identified protein binding sequences on the OPTN protein were blasted in either mouse or zebrafish protein databases and the likely orthologue was determined. The percent identity between the protein or partial sequences and the corresponding E-value are listed. For comparison, the analysis for the full-length sequence of IKBKG, the nearest paralogue to OPTN, is given. (DOCX) [file pone.0109922.s003.docx]

|  |  | | **% Identity** |  | **% Identity** |  |
| --- | --- | --- | --- | --- | --- | --- |
| **Binding site** | **OPTN residues** | **Reference** | **human-mouse** | **E-value** | **human-zebrafish** | **E-value** |
| Full length | 1-577 |  | 77% | 0.0* | 41% | 2e-108* |
| TBK1 | 1-127 | [98] | 82% | 7e-64* | 50% | 2e-17* |
| RAB8 | 141-209 | [59] | 77% | 2e-26* | 53% | 9e-08 |
| MAP1LC3B | 170-181 | [62] | 92% | 0.001 | 60% | 33 |
| GRM1A | 202-246 | [66] | 68% | 2e-05 | NR | NR |
| RAB11A | 324-375 | [67] | 94% | 7e-24* | 37% | 9e-04 |
| MYO6 | 412-520 | [51] | 83% | 1e-45* | 54% | 4e-24* |
| HTT | 411-461 | [59] | 80% | 2e-18* | 51% | 0.004 |
| CYLD | 412-577 | [50] | 84% | 6e-87* | 53% | 8e-47* |
| NRL | 423-577 | [99] | 87% | 3e-83* | 53% | 8e-47* |
| UB | 454-514 | [49] | 87% | 3e-21* | 55% | 5e-15* |
| **Binding site** | **IKBKG residues** | **Reference** | **human-mouse** | **E-value** | **human-zebrafish** | **E-value** |
| Full length | 1-419 |  | 87% | 0.0* | 46% | 3e-67* |
