# Supplementary material for: Loss of Optineurin In Vivo Results in Elevated Cell Death and Alters Axonal Trafficking Dynamics
Source: PLoS One. 2014 Oct 16;9(10):e109922. doi: 10.1371/journal.pone.0109922 (PMC4199637; doi:10.1371/journal.pone.0109922)
Supplement: Table S2 — Disease-associated residues in optineurin are conserved across species. Non-synonymous amino acid substitutions in OPTN were analyzed. For each mutation, the corresponding SNP designation, if applicable, and the amount of conservation of the similar region in mouse and zebrafish sequences are listed. Also listed are the disease association and reference for each mutation. Y = conservation of amino acid, N = no conservation, * = species has disease-associated residue. (DOCX) [file pone.0109922.s004.docx]

|  | |  | | **Conservation** | |  | |  | |
| --- | --- | --- | --- | --- | --- | --- | --- | --- | --- |
| **Residue substitution** | **SNP** | | **Mouse** | | **Zebrafish** | | **Disease association** | | **References** |
| H3Y |  | | Y | | N | | ALS | | [29] |
| P16A |  | | Y | | N | | ALS | | [29] |
| G23X |  | | Y | | Y | | ALS | | [25] |
| H26D |  | | N | | N | | glaucoma | | [7,14,18] |
| E50K | rs28939688 | | Y | | Y | | glaucoma | | [1,4,6,17,19] |
| A93P |  | | N | | Y | | ALS | | [27] |
| R96L |  | | Y | | Y | | ALS | | [22] |
| M98K | rs11258194 | | N *K | | N | | glaucoma | | [1,5,8,10,14,16,18–20,32] |
| E103D |  | | Y | | Y | | glaucoma | | [2] |
| V161M |  | | Y | | N | | ALS | | [31] |
| Q165X |  | | Y | | Y | | ALS | | [26,28] |
| T282P |  | | S | | S | | ALS | | [25] |
| Q314L | rs142812715 | | Y | | S | | ALS | | [25] |
| E322K | rs523747 | | Y | | Y | | glaucoma | | [3,32] |
| A336G |  | | Y | | N | | glaucoma | | [13] |
| A377T |  | | Y | | N | | glaucoma | | [13] |
| Q398X | rs267606928 | | Y | | S | | ALS | | [30,106] |
| I407T |  | | Y | | S | | glaucoma | | [9] |
| Q454E |  | | Y | | S | | ALS | | [28] |
| E478G | rs267606929 | | Y | | Y | | ALS | | [23,27,30] |
| H486R | rs373425395 | | Y | | Y | | glaucoma | | [2,16] |
| L500P |  | | Y | | N | | ALS | | [33,34] |
| R545Q | rs76554767 | | N *Q | | N *Q | | glaucoma | | [1,11,18] |
| K557T |  | | Y | | Y | | ALS | | [25] |
| L568S |  | | Y | | Y | | ALS | | [29] |
